# Clinical and genetic risk factors associated with neonatal severe hyperbilirubinemia: a case–control study based on the China Neonatal Genomes Project

**DOI:** 10.3389/fgene.2023.1292921

**Published:** 2024-01-11

**Authors:** Xiao Wang, Tiantian Xiao, Jin Wang, Bingbing Wu, Huijun Wang, Yulan Lu, Yaqiong Wang, Bin Chen, Liyuan Hu, Yun Cao, Rong Zhang, Guoqiang Cheng, Laishuan Wang, Zhihua Li, Xinran Dong, Lin Yang, Wenhao Zhou

**Affiliations:** ^1^ Center for Molecular Medicine, Children’s Hospital of Fudan University, National Children’s Medical Center, Shanghai, China; ^2^ Chengdu Women’s and Children’s Central Hospital, The Affiliated Women’s and Children’s Hospital, School of Medicine, University of Electronic Science and Technology of China (UESTC), Chengdu, China; ^3^ Department of Neonatology, Children’s Hospital of Fudan University, National Children’s Medical Center, Shanghai, China; ^4^ Department of Pediatric Endocrinology and Inherited Metabolic Diseases, Children’s Hospital of Fudan University, National Children’s Medical Center, Shanghai, China; ^5^ Guangzhou Women and Children’s Medical Center, Guangzhou Medical University, Guangzhou, China

**Keywords:** *UGT1A1* polymorphism, neonatal unconjugated hyperbilirubinemia, risk factors, generalized linear model, case-control analysis

## Abstract

**Objective:** We aimed to investigate the clinical and genetic risk factors associated with neonatal severe unconjugated hyperbilirubinemia.

**Methods:** This was a retrospective, 1:1 matched, case–control study. We included 614 neonates diagnosed with severe unconjugated hyperbilirubinemia (serum total bilirubin level ≥425 μmol/L or serum total bilirubin concentration that met exchange transfusion criteria) from the China Neonatal Genomes Project in Children’s Hospital of Fudan University. Clinical exome sequencing data were analyzed using a data analysis pipeline of Children’s Hospital of Fudan University. The factors associated with severe unconjugated hyperbilirubinemia were assessed using univariable and multivariable logistic regression analyses. Interaction analyses were examined between clinical and genetic risk factors.

**Results:** ABO/Rh incompatibility hemolysis (odds ratio [OR] 3.36, 95% confidence interval [CI] 2.32–4.86), extravascular hemorrhage (OR 2.95, 95% CI 2.24–3.89), weight loss (OR 5.46, 95% CI 2.88–10.36), exclusive breastmilk feeding (OR 3.56, 95% CI 2.71–4.68), and the homozygous mutant of UGT1A1 211G>A (OR 2.35, 95% CI 1.54–3.59) were all identified as factors significantly associated with severe unconjugated hyperbilirubinemia. The presence of UGT1A1 211G>A mildly increased the risk of severe unconjugated hyperbilirubinemia caused by ABO/Rh incompatibility hemolysis (OR 3.98, 95% CI 2.19–7.23), although the effect is not statistically significant.

**Conclusion:** ABO/Rh incompatibility hemolysis, extravascular hemorrhage, weight loss, exclusive breastmilk feeding, and the homozygous mutant of UGT1A1 211G>A were found to be risk factors for severe unconjugated hyperbilirubinemia. Clinical factors remain the most crucial and preventable determinants in managing severe unconjugated hyperbilirubinemia, with a minimal genetic contribution. The establishment of preconception care practices and the reinforcement of screening for the aforementioned risk factors are essential steps for preventing severe unconjugated hyperbilirubinemia.

## Introduction

Jaundice is a prevalent occurrence during the neonatal period and is typically a transient and benign phenomenon. However, with escalating total serum bilirubin (TSB) levels, an estimated 1 in 2,480 live births may manifest severe unconjugated hyperbilirubinemia (SHB), which is defined by TSB levels exceeding 342–427 μmol/L or meeting the criteria for exchange transfusion (Hyperbilirubinemia, 2004; Sgro et al., 2006; Kemper et al., 2022). These SHB neonates are at high risk for developing bilirubin-induced neurologic dysfunction (BIND). BIND arises when unbound bilirubin binds to brain tissue, triggering neurotoxicity (Bhutani and Johnson, 2009). Approximately 1.5% of term and late preterm neonates experiencing substantial hyperbilirubinemia may develop BIND (Dong et al., 2021). Despite its potential as a “never event,” BIND continues to pose a global burden (Bhutani et al., 2013; van der Geest et al., 2022).

Numerous studies have explored the levels of bilirubin in newborns and the corresponding risk factors for SHB or BIND. The early identification of these risk factors, combined with timely interventions such as phototherapy or exchange transfusion, has been shown to effectively reduce the incidence of BIND (Johnson et al., 2009; Wu et al., 2015). The management guidelines for hyperbilirubinemia in neonates with a gestation period of 35 or more weeks, as outlined by the American Academy of Pediatrics (AAP), are predicated on both TSB levels and associated risk factors (Kemper et al., 2022). Common risk factors associated with SHB or BIND included iso-immune hemolytic disease, G6PD deficiency, and infection (Han et al., 2015; Olusanya et al., 2015; Dong et al., 2021). However, a recent meta-analysis indicated that the risk factors of SHB varied in different populations from some low- and middle-income countries (LMICs), Asia, Europe, Australia, the United Kingdom, the United States, and Canada (Zhang et al., 2021).

In addition to the prevalent clinical factors, the current management guidelines incorporate limited consideration for genetic factors. There is a scarcity of large-scale studies investigating the genetic factors associated with SHB (Mei et al., 2022). Among the genetic factors, the impact of *UGT1A1* gene polymorphisms in SHB has emerged as a focal point of investigation. *UGT1A1* polymorphism refers to the presence of genetic variations (single-nucleotide polymorphisms [SNPs], insertions, deletions, or other types of mutations) in the *UGT1A1* gene among individuals within a population. To date, there are two major types of *UGT1A1* gene polymorphisms associated with neonatal hyperbilirubinemia, namely, alterations in the TATA box of the promoter, specifically changes in the repeat number of TA dinucleotide {UGT1A1*28 [A(TA)7TAA]}, and mononucleotide substitution mutations within the coding region [UGT1A1*6 (c.211G>T)]. However, limited evidence from studies suggests a direct association between *UGT1A1* gene polymorphisms and the occurrence of SHB (Nguyen et al., 2020). Most of related studies only enrolled a small sample size or focused on investigating jaundice in neonates, using non-jaundiced individuals as the control group (Mehrad-Majd et al., 2019). Theoretically, SHB is not only attributed to an elevated production of serum bilirubin but also to the metabolic dysfunction of UGT1A1 (Olusanya et al., 2015). However, little is known regarding the interaction effect of *UGT1A1* gene polymorphism and other common factors in neonates with SHB. Therefore, it is necessary to gain more insights into preventable factors which contribute to the development of SHB in a large population.

Accordingly, we aim to investigate the association of common clinical factors and *UGT1A1* gene polymorphisms with SHB and its complications in a large population based on the China Neonatal Genomes Project and further explore the interaction effect between the *UGT1A1* gene polymorphism and other clinical factors on neonates with SHB.

## Methods

### Study design and population

**FIGURE 1:**
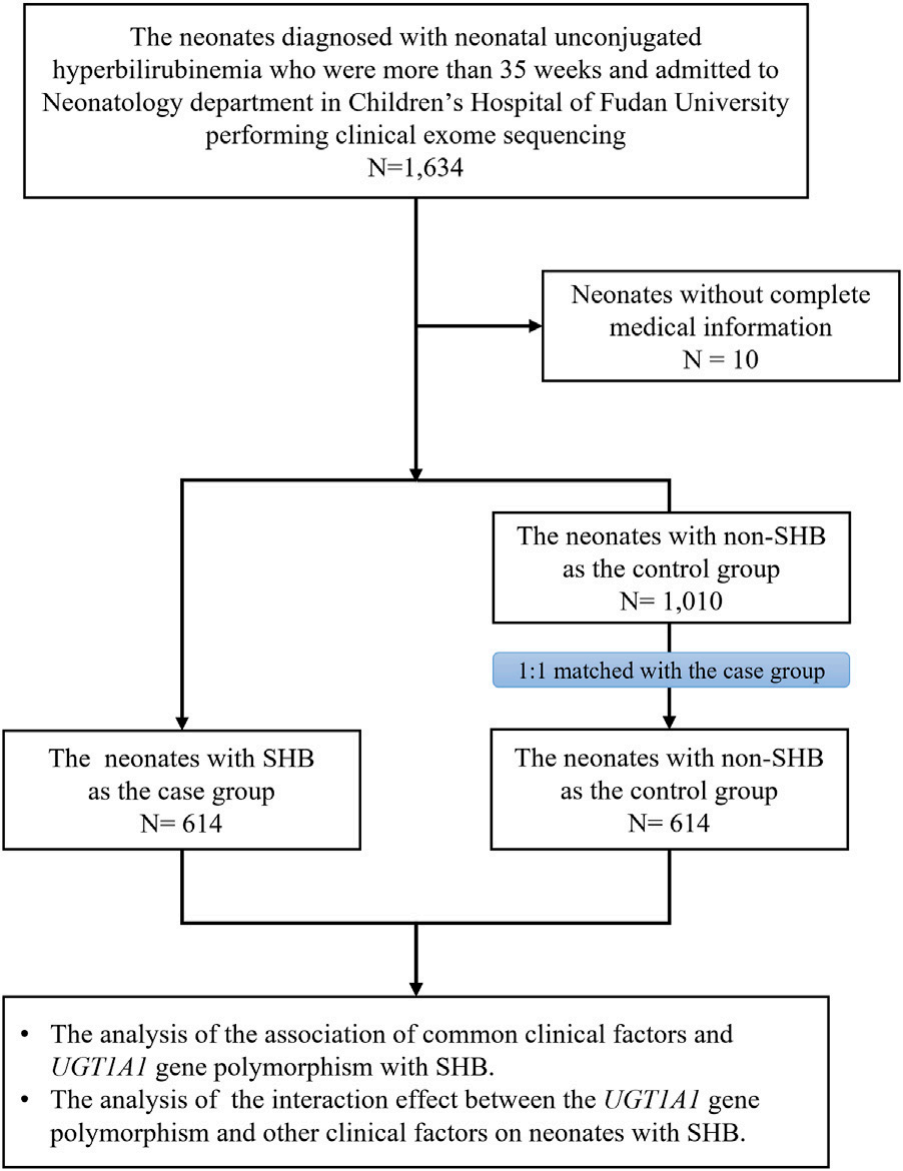
Study workflow in the neonates with unconjugated hyperbilirubinemia. Univariate and multivariable logistic models were used for the association analyses. SHB, severe hyperbilirubinemia; the neonates who had TSB level ≥425 μmol/L or TSB concentration that met exchange transfusion criteria according to the AAP guidelines were defined as the severe group. Non-SHB: non-severe hyperbilirubinemia, which is defined as neonates with TSB of less than 256 μmol/L or TSB concentration that did not meet exchange transfusion criteria.

This is a case–control study (Figure 1). We collected patients diagnosed with neonatal unconjugated hyperbilirubinemia who were admitted to the neonatology department in Children’s Hospital of Fudan University and also enrolled in the China Neonatal Genomes Project. The exclusion criteria are as follows: neonates 1) whose gestational age (GA) was less than 35 weeks; 2) who did not undergo clinical exome sequencing; and 3) whose medical information was incomplete. The neonates who had a TSB level ≥425 μmol/L or TSB concentration that met exchange transfusion criteria according to the AAP guidelines (Hyperbilirubinemia, 2004) were defined as the severe group, while the non-severe group was defined as those neonates with a TSB of less than 256 μmol/L or TSB concentration that did not meet exchange transfusion criteria (Hyperbilirubinemia, 2004).

The study received approval from the Ethics Committee of Children’s Hospital of Fudan University under the reference CHFudanU_NNICU11. Written informed consent was obtained from the parents of each neonate, ensuring adherence to ethical standards and respecting the principles of research integrity and participant autonomy.

### Clinical variables and definition

The baseline information included gender, GA (days), birth weight (BW, in grams), days to peak bilirubin post-birth, and total bilirubin level (TSB, denoting the highest value among all TSB results during hospitalization, measured in μmol/L). Subsequent to hospitalization, we collected additional clinical and genetic features, including ABO/Rh incompatibility hemolysis (evaluated through Coombs test), G6PD deficiency (indicated by G6PD enzyme activity of less than 2.2 units/gram of hemoglobin), infection (sepsis and urinary tract infection), extravascular hemorrhage (cephalohematoma or intracranial hemorrhage), polycythemia, *UGT1A1-*positive [patients who underwent clinical exome sequencing as part of the China Neonatal Genomes Project, which identified pathogenic/likely pathogenic variants of *UGT1A1* that meet diagnostic criteria according to the ACMG guidelines, such as NM_000463.3 (*UGT1A1*): c.1002del (p.Trp335fs) and NM_000463.3 (*UGT1A1*): c.1006C>T (p.Arg336Trp)], congenital hypothyroidism, abnormal weight loss, delayed passage of meconium, breastfeeding (exclusive or mixed), and other genetic diagnoses (patients who underwent clinical exome sequencing as part of the China Neonatal Genomes Project, which identified pathogenic/likely pathogenic variants in genes other than *UGT1A1* that meet diagnostic criteria according to the ACMG guidelines, including genes *ABCC8*, *ARID1B*, *CDK13*, *EARS2*, *KCNQ2*, *RARS2*, and *STXBP2*). Among these variables, gender, weight loss, hemorrhage, breastmilk feeding, hemolysis, G6PD deficiency, infections, polycythemia, being *UGT1A1-*positive, delayed passage of meconium, genetic diagnoses, and hypothyroidism were categorical.

Auditory neuropathy spectrum disorder (ANSD) is defined as the absent or abnormal morphology of a brainstem auditory evoked response (BAER) waveform at 80-decibel click intensity, alongside normal outer hair cell function at discharge. Abnormalities in magnetic resonance imaging (MRI) of the brain are defined as bilateral injury to the globus pallidus and subthalamic nucleus at discharge (Li Q. et al., 2021b).

### Sequencing, variant calling, and *UGT1A1* polymorphism identification

Clinical exome sequencing was performed on patients following the protocol described in our previous studies (Yang et al., 2019). In brief, DNA was extracted from patients’ peripheral blood according to the manufacturer’s instructions using the Thermo Fisher Scientific (Shanghai, China) KingFisher LabServ kit. Genomic DNA was enriched using the Agilent ClearSeq Inherited Disease panel kit (Santa Clara, CA, United States) or NanoWES Human Exome Kit (Berry Genomics, Beijing, China). DNA libraries were sequenced on the Illumina HiSeq 2000/2500 or NovaSeq 6000 platform (San Diego, CA, United States) to yield 150-bp paired-end sequencing reads. Only overlapped sequencing regions of the above two captures, single-nucleotide variants (SNVs), and small insertions and deletions (InDels) were included for downstream analysis. Raw reads were mapped to *Homo sapiens* genome assembly GRCh37 with BWA (Li and Durbin, 2009) and further processed by GATK3 (Van der Auwera et al., 2013) for genotyping. A total of 151 high-quality SNVs and InDels in *UGT1A1* (chr2: 234668915-234681206, where the promoter region was not considered because of capture limit) were obtained. Variation sites were further annotated with Ensembl Variant Effect Predictor (McLaren et al., 2016). For sites annotated with multiple transcripts, only canonical transcripts of *UGT1A1* (NM_000463.3) were kept with the predicted effects of each allele.

Polymorphisms with an allele frequency greater than 1% in the cohort were considered SNP sites and InDels, as given in Supplementary Table S1. These variants were assumed to potentially influence TSB and were categorized according to three genotypes: same as reference, heterozygous, and homozygous.

The variation data have been deposited in the Genome Variation Map (Li C. et al., 2021a) in the National Genomics Data Center (Yongbiao et al., 2022), China National Center for Bioinformation/Beijing Institute of Genomics, Chinese Academy of Sciences, under accession number GVM000666 that can be publicly accessible at http://bigd.big.ac.cn/gvm/getProjectDetail?project=GVM000666.

### Sample size calculation

**FIGURE 2:**
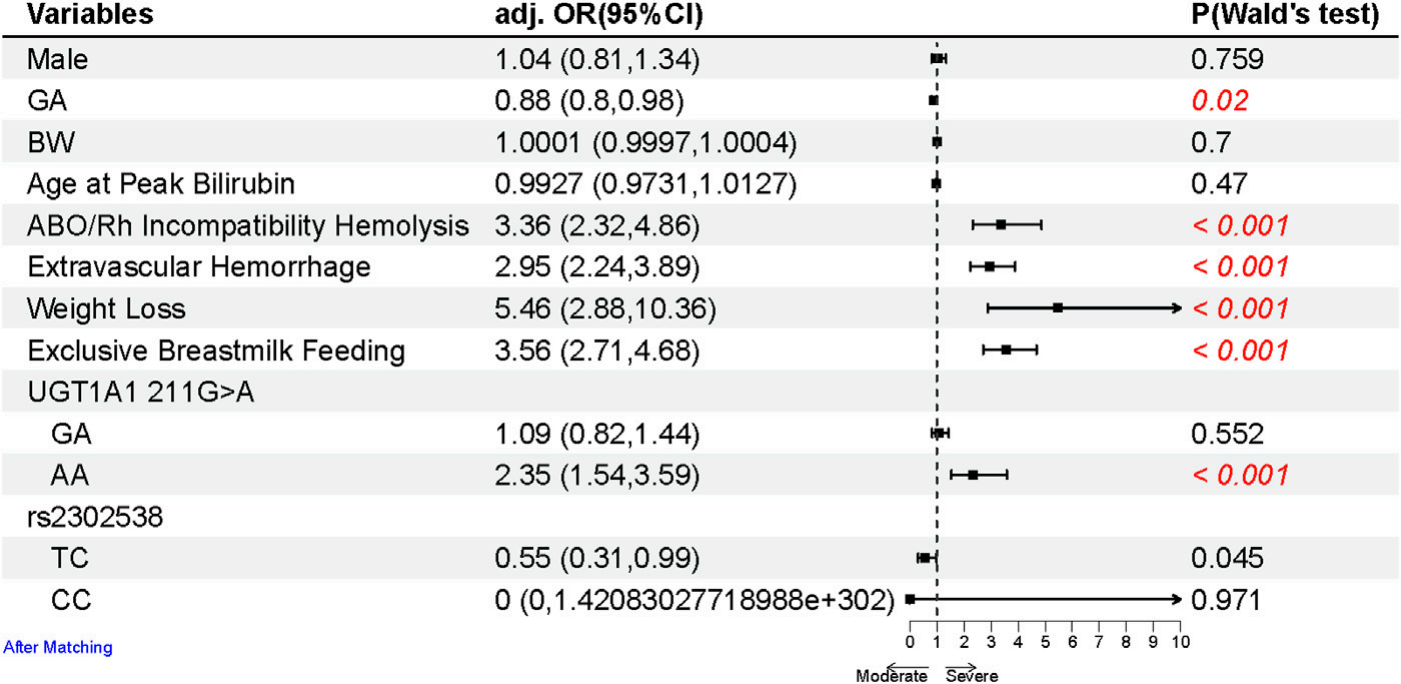
Forest plot of the risk factors for the neonates with SHB after matching sex, GA, BW, and age at peak bilirubin (due to different age groups having varying criteria of SHB). Multivariate logistic regression analyses between the SHB and control groups were conducted using binomial generalized linear models (glm) and described as OR with 95% CIs. All statistical tests were two-tailed, with a significance level at 0.05. *p*-values less than 0.05 are highlighted in red.

The sample size calculation is based on the severe and non-severe hyperbilirubinemia groups of equal size, with levels of confidence = 95% and power = 90%. The expected homozygous UGT1A1 211G>A allele frequency in the control groups is set to 37%, with assumed odds ratio (OR) at 2.35 (Figure 2). For these inputs, a sample size of at least 113 subjects per group is required from an online sample size calculator (Sergeant, 2018) (https://epitools.ausvet.com.au/casecontrolss), which is far below the real sample inclusion.

### Statistical analysis

Statistical analyses were performed using R4.1.1. Univariate analyses in baseline characteristics were conducted between severe (the case group) and non-severe groups (the control group) using the chi-squared test for categorical variables and Student’s t-test for continuous variables (Panos and Mavridis, 2020). Multivariate logistic regression analyses between the two groups were examined using binomial generalized linear models (glm) with the default logit link function and described as OR with 95% confidence intervals (CIs). All statistical tests were two-tailed, with a significance level at 0.05. To create case and control groups balanced on included covariates, all studied neonates between severe (the case group) and non-severe groups (the control group) were matched for gender, GA, BW, and age at peak bilirubin (due to different age groups having varying criteria of SHB), using the 1:1 nearest neighbor matching for propensity scores estimated with logistic regression (Stuart et al., 2011).

We conducted interaction analyses between *UGT1A* polymorphism and clinical risk factors including ABO/Rh incompatibility, hemolysis, hemorrhage, weight loss, and exclusive breastfeeding. This investigation incorporated both additive and multiplicative scales, integrating a product term of the genetic factor with each of the four clinical factors into the linear regression models. We calculated three statistics, namely, relative excess risk (RERI), attributable proportion (AP), and synergy index (SI), along with corresponding *p*-values and 95% CIs (Alli, 2021).

## Results

### Clinical characteristics of the study population

A total of 614 neonates constituted the SHB group (the case group), with an average of (TSB level of 428.32 (84.53) μmol/L. Through meticulous 1:1 matching based on gender, GA, BW, and age at peak bilirubin, a corresponding control group of 614 neonates was established to mitigate confounding variables and enhance study efficiency (Rose and Van der Laan, 2009). The clinical characteristics of the matched cohorts are given in Table 1. Within the matched cohort, significant differences emerged in the prevalence of ABO/Rh incompatibility hemolysis (18.9% vs. 10.3%), extravascular hemorrhage (40.9% vs. 21.8%), congenital hypothyroidism (3.9% vs. 2.9%), weight loss (11.4% vs. 2.1%), delayed passage of meconium (0.8% vs. 0.2%), and exclusive breastmilk feeding (55.5% vs. 29.5%), with higher proportions observed among SHB neonates (the case group). The frequency distribution of *UGT1A1* polymorphisms is given in Supplementary Table S1 and Supplementary Figure S1. There is a significantly higher allele frequency of UGT1A1 211G>A in SHB neonates (44.0% vs. 38.7%).

**TABLE 1:** Baseline characteristics of matched severe hyperbilirubinemia cohorts.

Variable		Control (n = 614)	Case (n = 614)	*p* ^a^
Male (%)		340 (55.4)	333 (54.2)	0.731
GA^b^ [mean (SD^c^)]		38.50 (1.54)	38.40 (1.36)	0.2
BW^d^ [mean (SD)]		3,293.80 (437.30)	3,285.42 (431.63)	0.735
Age at peak bilirubin^e^ [mean (SD)]		6.49 (8.47)	6.93 (4.39)	0.25
Total bilirubin level^f^ [mean (SD)]		213.86 (78.27)	428.32 (84.52)	<0.001**
Risk factors^g^				
**Clinical factors**	ABO/Rh incompatibility hemolysis (%)	63 (10.3)	116 (18.9)	<0.001**
	Infection (%)	156 (25.4)	142 (23.1)	0.387
	Extravascular hemorrhage (%)	134 (21.8)	251 (40.9)	<0.001**
	Polycythemia (%)	7 (1.1)	13 (2.1)	0.26
	Congenital hypothyroidism (%)	18 (2.9)	24 (3.9)	0.432
	Weight loss (%)	13 (2.1)	70 (11.4)	<0.001**
	Delayed passage of meconium (%)	1 (0.2)	5 (0.8)	0.22
	Exclusive breastmilk feeding (%)	181 (29.5)	341 (55.5)	<0.001**
**Genetic factors**	G6PD deficiency (%)	2 (0.3)	7 (1.1)	0.181
	*UGT1A1* positive^h^ (%)	0 (0.0)	1 (0.2)	1
	Other genetic diagnoses^i^ (%)	1 (0.2)	7 (1.1)	0.076
*UGT1A1* polymorphism^j^	UGT1A1 211G>A (%)	238 (38.7)	270 (44)	0.001**
	GG	376 (61.2)	344 (56.0)	
	GA	190 (30.9)	180 (29.3)	
	AA	48 (7.8)	90 (14.7)	
	rs139595073 (%)	33 (5.4)	35 (5.7)	0.952
	(T)_22_ (T)_22_	581 (94.6)	579 (94.3)	
	(T)_22_ del/dup(T)_n_	13 (2.1)	13 (2.1)	
	del/dup(T)_n_ del/dup(T)_n_	20 (3.3)	22 (3.6)	
	rs4148327 (%)	39 (6.4)	38 (6.2)	0.114
	TT	575 (93.6)	576 (93.8)	
	TC	39 (6.4)	34 (5.5)	
	CC	0 (0.0)	4 (0.7)	
	rs2302538 (%)	44 (7.1)	23 (3.7)	0.019*
	TT	570 (92.8)	591 (96.3)	
	TC	42 (6.8)	23 (3.7)	
	CC	2 (0.3)	0 (0.0)	
	UGT1A1 1091C>T (%)	47 (7.7)	33 (5.4)	0.134
	CC	567 (92.3)	581 (94.6)	
	CT	47 (7.7)	32 (5.2)	
	TT	0 (0.0)	1 (0.2)	
	rs4663334 (%)	11 (1.8)	12 (2.0)	0.526
	CC	603 (98.2)	602 (98.0)	
	CT	8 (1.3)	6 (1.0)	
	TT	3 (0.5)	6 (1.0)	

*p<0.05.

**p < 0.01.

^a^ Univariate analyses in baseline characteristics were conducted between severe (the case group) and non-severe groups (the control group) using the chi-square test for categorical variables and Student’s t-test for continuous variables.

^b^ GA: gestational age (weeks).

^c^ SD: standard deviation.

^d^ BW: birth weight (g).

^e^ Age at peak bilirubin: days to peak bilirubin after birth (days).

^f^ Total bilirubin level (μmol/L).

^g^ The other values in the Risk Factors section refer to the number of patients under each respective factor.

^h^ *UGT1A1* positive: number of patients diagnosed with pathogenic/likely pathogenic variants of *UGT1A1* according to the ACMG guidelines.

^i^ Other genetic diagnoses: number of patients diagnosed with pathogenic/likely pathogenic variants in genes other than *UGT1A1* according to the ACMG guidelines.

^j^ UGT1A1 Polymorphism: number of patients with each respective genotype. Each polymorphism is categorized into three genotypes: reference, heterozygous, and homozygous, e.g., GG, GA, and AA. Values following the sub-term [e.g., UGT1A1 211G>A (%)] represent the sum of heterozygous and homozygous counts.

### The clinical factor and *UGT1A1* gene polymorphisms were associated with severe neonatal hyperbilirubinemia

To identify risk factors for SHB, a comparative analysis between the case and control groups was conducted using a multivariable logistic regression model (Figure 2). Neonates with SHB exhibited a higher likelihood of ABO/Rh incompatibility hemolysis (OR 3.36, 95% CI 2.32–4.86), extravascular hemorrhage (OR 2.95, 95% CI 2.24–3.89), weight loss (OR 5.46, 95% CI 2.88–10.36), and exclusive breastmilk feeding (OR 3.56, 95% CI 2.71–4.68).

Regarding *UGT1A1* polymorphisms (including UGT1A1 211G>A, among others; Supplementary Table S1), they were considered potential risk factors for SHB and were categorized into three types (same as the reference, heterozygous, and homozygous) based on the genotype, as described in the *Methods* section. Of the six polymorphisms, the homozygous mutant of UGT1A1 211G>A emerged as a significant contributor to the increased risk of developing SHB (OR 2.35, 95% CI 1.54–3.59).

### The performance of the predictive model based on the clinical and genetic risk factors

**FIGURE 3:**
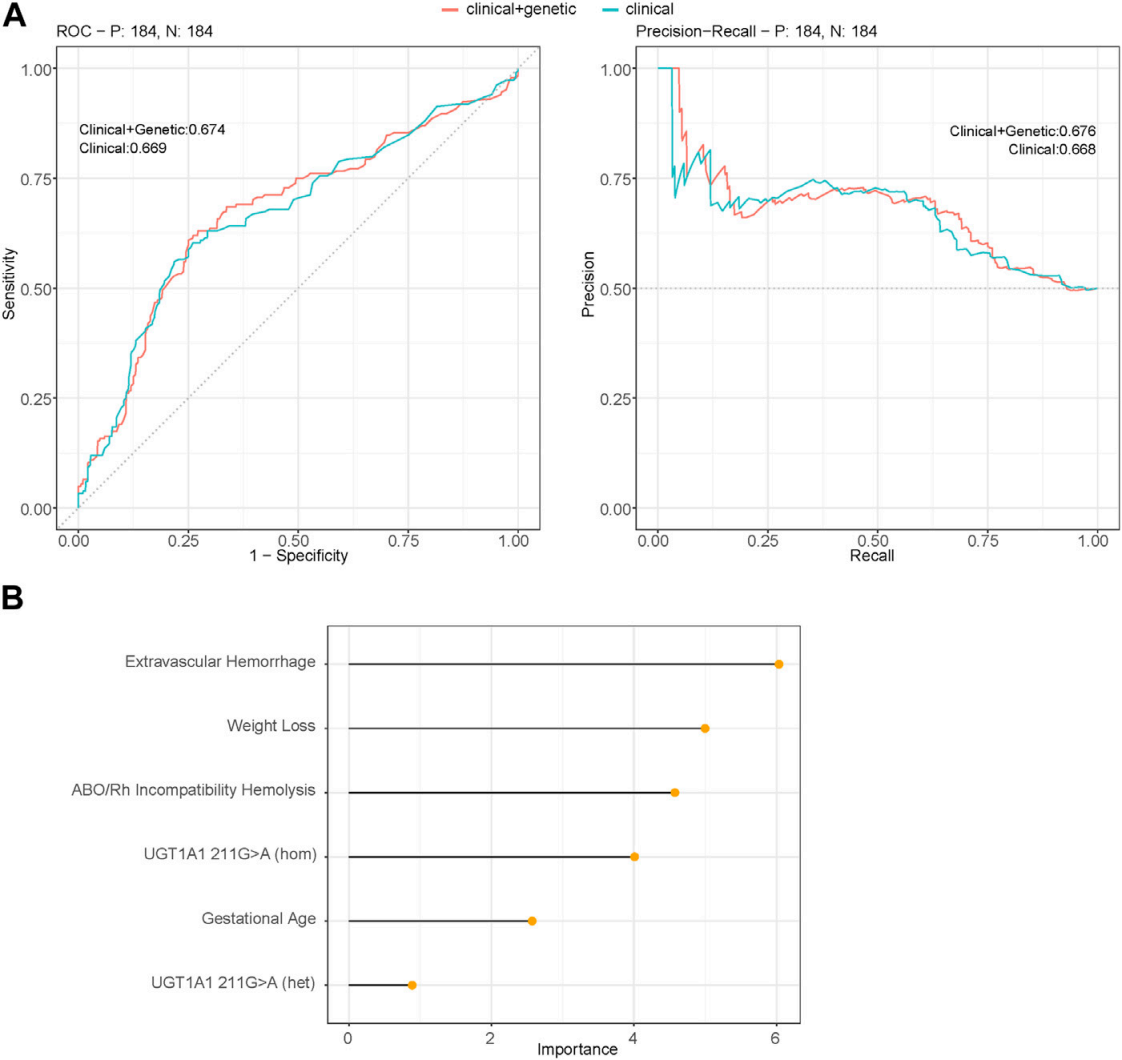
**(A)** Performance of the multivariable logistic regression models using binomial generalized linear models (glm) between the SHB group and the non-severe group (control group) that predict the severity of hyperbilirubinemia. The figure represents the area under receiver operating characteristic curve (AUROC) and the area under the precision-recall curve (AUPRC) obtained for the testing set of 30% of the cohort using significant variables. Clinical + genetic model includes both clinical factors: GA, ABO/Rh incompatibility hemolysis, extravascular hemorrhage, weight loss, and genetic risk variables: UGT1A1 211G>A; the clinical model uses only clinical factors (AUROC DeLong test between two predicted models: 0.689). **(B)** Variable importance of the prediction model incorporating both clinical factors and genetic factor 211G>A as predictors (model: clinical + genetic).

To validate the results from the multivariable logistic regression model, we conducted a robust validation process by partitioning the severe hyperbilirubinemia cohorts into 70% training and 30% testing sets. In this validation approach, we employed a logistic regression model, using potential risk factors as predictors to estimate the performance of the prediction model. The predictors incorporated in the model comprised the baseline features: GA and clinical risk factors including ABO/Rh incompatibility hemolysis, extravascular hemorrhage, and weight loss (exclusive breastmilk feeding eliminated due to its significant correlation with weight loss, chi-squared test, *p* = 1.43e−09). Additionally, the genetic risk variable: UGT1A1 211G>A was included, with the variant categorized based on the genotype: GG (same as reference), GA (heterozygous), and AA (homozygous). This comprehensive set of predictors aimed to capture the diverse dimensions contributing to the risk of severe hyperbilirubinemia. The predictive model demonstrated a strong performance on the SHB testing set (AUC: 0.674, 95% CI: 0.618–0.730, specificity: 0.620 and sensitivity: 0.739). Notably, this performance was marginally superior to that of the prediction model without genetic variables (AUC: 0.669, 95% CI: 0.614–0.725), although the difference in AUCs between the two models was not statistically significant (DeLong test: 0.689) (Figure 3A). Figure 3B illustrates the variable importance of the predicted model, with extravascular hemorrhage contributing the most, followed by weight loss, ABO/Rh incompatibility hemolysis, and the homozygous mutant of UGT1A1 211G>A. The results suggest that while the heterozygous mutant of 211G>A serves as a risk factor, it contributes the least to the predictive model.

### The interaction effect of the significant *UGT1A1* gene polymorphisms and clinical factors associated with severe hyperbilirubinemia

The homozygous UGT1A1 211G>A genotype emerged as the only significant genetic risk factor for SHB. To delve deeper into its impact, we explored the interaction effect between the UGT1A1 211G>A genotype and clinically significant risk factors (Table 2). The reference category was assigned to individuals without the 211G>A genotype and without hemolysis. The ORs for the UGT1A1 211G>A genotype in conjunction with ABO/Rh incompatibility hemolysis were 3.98 (95% CI 2.19–7.23), highlighted in bold in Table 2. This value is significantly higher than 1 and surpasses both the ORs of 211G>A genotype alone (OR, 1.36, 95% CI 1.04–1.78) and hemolysis alone (OR, 3.34, 95% CI 2.14–5.22). Although the degree of enhanced interaction was not statistically significant (measured by RERI, AP, and SI), the combined effect on the OR scale of the UGT1A1 211G>A genotype and ABO/Rh incompatibility hemolysis still suggests an intensified influence on the risk of SHB. For other clinical factors, no interaction effect was observed between the UGT1A1 211G>A genotype and extravascular hemorrhage, weight loss, and breastmilk feeding.

**TABLE 2:** Interaction of ABO/Rh incompatibility and the UGT1A1 211G>A genotype on the risk of severe hyperbilirubinemia^a^.

	UGT1A1 211G>A absent	UGT1A1 211G>A present
	OR [95% CI]	OR [95% CI]
ABO/Rh incompatibility hemolysis absent	1 [reference]	1.36 [1.04, 1.78] *p* = 0.026
ABO/Rh incompatibility hemolysis present	3.34 [2.14, 5.22] *p* = 1.258e−07	**3.98 [2.19, 7.23] p = 7.192e−06**
Multiplicative scale	0.88 [0.42, 1.84] *p* = 0.733	
RERI	0.29 [−2.34, 2.91] *p* = 0.416	
AP	0.07 [−0.55, 0.7] *p* = 0.411	
SI	1.11 [0.45, 2.74] *p* = 0.107	

^a^ The interaction analyses integrated a product term of the genetic factor with each of the four clinical factors into the linear regression models. ORs are adjusted for GA, extravascular hemorrhage, weight loss, and exclusive breastmilk feeding.

The bold value refers to the joint Odds Ratio (OR) and its corresponding confident interval and *p*-value for the UGT1A1 211G>A genotype and ABO/Rh incompatibility hemolysis. This value is significantly higher than 1 and surpasses both the OR of 211G>A alone (1.36) and hemolysis alone (3.34). This indicates the enhanced interaction effect between these two variables.

### The risk factors associated with the abnormality of MRI and ANSD among the neonates with severe hyperbilirubinemia

In our further investigation, we explored the risk factors associated with the abnormalities in MRI and ANSD among the SHB neonates in this cohort. After matching for GA, BW, and sex, we identified 614 SHB neonates, among whom only 35 exhibited abnormal MRI results and 44 were diagnosed with ANSD. Upon comparison with the neonates from the matched control group, no significant findings were observed in the SHB neonates with abnormal MRI or ANSD (Supplementary Figure S2).

## Discussion

Neonatal unconjugated hyperbilirubinemia stands out as one of the most prevalent conditions, accounting for around 50% of neonatal rehospitalization (Mei et al., 2022). Elevated serum bilirubin levels pose a significant risk for BIND, and vigilant monitoring coupled with appropriate interventions can effectively mitigate the risk of BIND. A population-based study investigated the global incidence of severe neonatal jaundice, defining it as hyperbilirubinemia associated with BIND, exchange transfusions, or jaundice-related death (Slusher et al., 2017). Notably, Southeast Asia exhibited the second-highest incidence of SHB at 251.3 occurrences per 10,000 live births. In contrast, the lowest incidence was reported in the Americas (4.4 occurrences per 10,000 live births) and Europe (3.7 occurrences per 10,000 live births). These findings underscore the persistent and significant health burden posed by SHB, particularly in the Asian region.

In our comprehensive study, we conducted a systematic investigation of both established clinical risk factors and UGT1A1 gene polymorphisms associated with SHB. Aligning with earlier research findings (Mehrad-Majd et al., 2019; Nguyen et al., 2020), our study identified four significant clinical factors, ABO/Rh incompatibility hemolysis, extravascular hemorrhage, weight loss, and exclusive breastmilk feeding. An observational study in Denmark exploring risk factors for extreme SHB in neonates with GA ≥ 35 weeks and TSB ≥ 450 μmol/L found that ABO incompatibility (*n* = 58), cephalohematoma (*n* = 14), G6PD deficiency (*n* = 6), and hypothyroidism (*n* = 4) were several explanatory factors for SHB (Sgro et al., 2006). Notably, our study revealed a strong association between exclusive breastmilk feeding and hyperbilirubinemia. Current AAP guidelines classified breastmilk-related jaundice into two categories according to the onset time of jaundice and complications, namely, breastfeeding jaundice and breastmilk jaundice (Kemper et al., 2022). In our study, we found exclusive breastmilk feeding increased the risk for developing SHB, and our further analysis showed that exclusive breastmilk feeding is significantly correlated with weight loss (chi-squared test, *p* = 1.43e−09), which is perceived as the result of suboptimal intake, a risk factor for developing SHB (Kemper et al., 2022). Hence, our findings suggest that neonates who are exclusively breastfed in the first few days should be closely monitored for factors such as weight, stool frequency, urine output volume, and jaundice levels as a preventive measure against the development of SHB in clinical practice.

Genetic polymorphisms that alter enzyme expression have been identified in the *UGT1A1* gene. This gene is responsible for the production of UDP-glucuronosyltransferases, the sole enzymes capable of glucuronidating bilirubin. This crucial enzymatic process converts the toxic unconjugated bilirubin into its non-toxic, conjugated form, rendering it soluble and facilitating its removal from the body. Although a consensus has been reached on the role of UGT1A1 polymorphisms in neonatal unconjugated hyperbilirubinemia, their association with SHB remains uncertain. Previous studies conducted in Asia, often comparing neonates with hyperbilirubinemia to a control group without hyperbilirubinemia, have identified UGT1A1*6 (c.211G>T) and UGT1A1*28 [A(TA)7TAA] as two common variants associated with hyperbilirubinemia (Slusher et al., 2017; Mei et al., 2022). Consistent with previous research findings (Yang et al., 2016; Yu et al., 2020), our study identified UGT1A1*6 (c.211G>T) as one of the common variants, prevalent in 44.0% of SHB neonates. Furthermore, our cohort revealed additional common and rare polymorphism sites (Supplementary Figure S1; Supplementary Table S1). Distinct from some previous studies (Yang et al., 2016; Yang et al., 2021), our results uniquely showed that the homozygous mutant of UGT1A1 211G>A heightened the risk of SHB (Nguyen et al., 2020). However, the heterozygous mutation of UGT1A1 211G>A did not significantly aggravate illness in neonates (Yu et al., 2020).

Currently, few studies have investigated the interaction effect of the UGT1A1 211G>A and clinical risk factors (Yang et al., 2021). Consistent with previous studies (Yu et al., 2020; Yang et al., 2021), our findings suggest that the presence of UGT1A1 211G>A may mildly aggregate the severity of hyperbilirubinemia caused by ABO/Rh incompatibility, although the interaction effect was not significant, whereas UGT1A1 211G>A did not increase the risk of SHB combined with other clinical factors, consistent with findings in a study from Turkey (Halis et al., 2017). The other study from Japan indicated that UGT1A1 211G>A served as a risk factor for neonatal hyperbilirubinemia only in infants with inadequate breastfeeding (Sato et al., 2013). Additional research is needed to elucidate the molecular and physiological mechanisms that contribute to this effect.

We further proved our findings by constructing a predictive model for SHB. In the prediction phase, we used factors identified as significant in the multivariate analysis (GA, ABO/Rh incompatibility hemolysis, extravascular hemorrhage, weight loss, and breastmilk feeding) to predict the likelihood of SHB. The area under the curve (AUC) in the testing set showed a robust performance. However, when incorporating *UGT1A1* polymorphisms into the model, the AUC in the testing set showed no significant difference. In general, *UGT1A1* polymorphisms make only a modest contribution to the prediction. Therefore, for the severity of neonatal unconjugated hyperbilirubinemia in early life, the clinical factors are the predominant contributors (Sato et al., 2013).

In our study, we discussed the short-term outcomes associated with SHB. Previous studies indicated that some clinical risk factors, such as Rh incompatibility, sepsis, and low admission weight, rather than TSB levels, were associated with BIND (Gamaleldin et al., 2011; Amin et al., 2016). However, our findings showed that SHB-related MRI abnormalities and ANSD were not associated with TSB levels, the onset age of peak bilirubin, or other clinical factors. It is essential to note that due to limited data, some results did not reach statistical significance. Consequently, further studies are warranted to comprehensively investigate the risk factors associated with SHB.

## Limitations

Several limitations should be acknowledged in this study. First, although a meta-analysis has elucidated the association of neonatal hyperbilirubinemia with *UGT1A1* promoter variations (Yu et al., 2015), we did not explore the promoter region in our study due to the unavailability of clinical exome sequencing data for reanalysis. Second, our analysis focused solely on the *UGT1A1* gene. Considering additional genes in the assessment could provide a more comprehensive understanding of the genetic factors contributing to unconjugated hyperbilirubinemia in newborns. Third, the study exclusively included neonates from a single hospital in China, warranting the need for a larger multi-center study in future research endeavors.

## Conclusion

In this large cohort study based on the China Neonatal Genomes Project, ABO/Rh incompatibility hemolysis, extravascular hemorrhage, weight loss, exclusive breastfeeding, and UGT1A1 211G>A homozygosity emerged as predominant factors associated with the development of SHB. In addition, UGT1A1 211G>A was found to mildly exacerbate the severity of hyperbilirubinemia induced by ABO/Rh incompatibility, although the effect is not statistically significant.

In conclusion, clinical factors remain the most crucial and preventable determinants in managing jaundice, with a minimal genetic contribution. These findings emphasize that screening clinical factors remains paramount in the proactive prevention of SHB.

## Data Availability

The datasets presented in this study can be found in online repositories. The variation data reported in this paper have been deposited in the Genome Variation Map [1] in the National Genomics Data Center [2], China National Center for Bioinformation/Beijing Institute of Genomics, Chinese Academy of Sciences, under accession number GVM000666 that can be publicly accessible at http://bigd.big.ac.cn/gvm/getProjectDetail?project=GVM000666. 1) Genome Variation Map: a worldwide collection of genome variations across multiple species. Nucleic Acids Res 2021, 49(D1):D1186-D1191. [PMID = 33170268]. 2) Database Resources of the National Genomics Data Center, China National Center for Bioinformation in 2021. Nucleic Acids Res 2021, 49(D1):D18-D28. [PMID = 33175170].
