# Supplementary material for: Clinical and genetic risk factors associated with neonatal severe hyperbilirubinemia: a case–control study based on the China Neonatal Genomes Project
Source: Front Genet. 2024 Jan 11;14:1292921. doi: 10.3389/fgene.2023.1292921 (PMC10808734; doi:10.3389/fgene.2023.1292921)
Supplement: Supplementary file 1 [file DataSheet1.docx]

**Supplemental Content**

**supTable1.** *UGT1A1* polymorphisms in SHB cohorts.

**supFigure1.** Allele counts (the left x-axis) and frequencies (the right x-axis) of top 20 of *UGT1A1* polymorphisms. Different color denotes specific predicted effects of each allele on its corresponding canonical transcript annotated by VEP.

**supFigure2.** Forest plot of the risk factors for severe hyperbilirubinemia related the abnormality of magnetic resonance imaging and auditory neuropathy spectrum disorder.

**a.** Forest plot of the risk factors for severe hyperbilirubinemia related the abnormality of magnetic resonance imaging.

**b.** Forest plot of the risk factors for severe hyperbilirubinemia related the auditory neuropathy spectrum disorder.

**supTable1.** *UGT1A1* polymorphisms in SHB cohorts.

| Variants | Genomic coordinate^1^ | Reference allele | Alternative allele | HGVS |
| --- | --- | --- | --- | --- |
| rs4148323 (UGT1A1 211G>A) | chr2: 234669144 | G | A | NM_000463.3:exon1:  c.211G>A(p.G71R) |
| rs139595073 | chr2: 234677228 | (T)_22_ | del(T)_n_ /dup(T_)n_ /ins(T)_n_ /ins(T)_n_ | NM_000463.2:c.1304+152_1304+165del etc. |
| rs4148327 | chr2: 234675826 | T | C | NM_000463.3:c.996+15T>C |
| rs2302538 | chr2: 234676413 | T | C | NM_000463.3:c.997-82T>C |
| rs34946978 (UGT1A1 1091C>T) | chr2: 234676872 | C | T | NM_000463.3:exon4:  c.1091C>T(p.P364L) |
| rs4663334 | chr2: 234674924 | C | T | NM_000463.3:c.865-756C>T |

^1^Reference Build: Homo sapiens genome assembly GRCh37


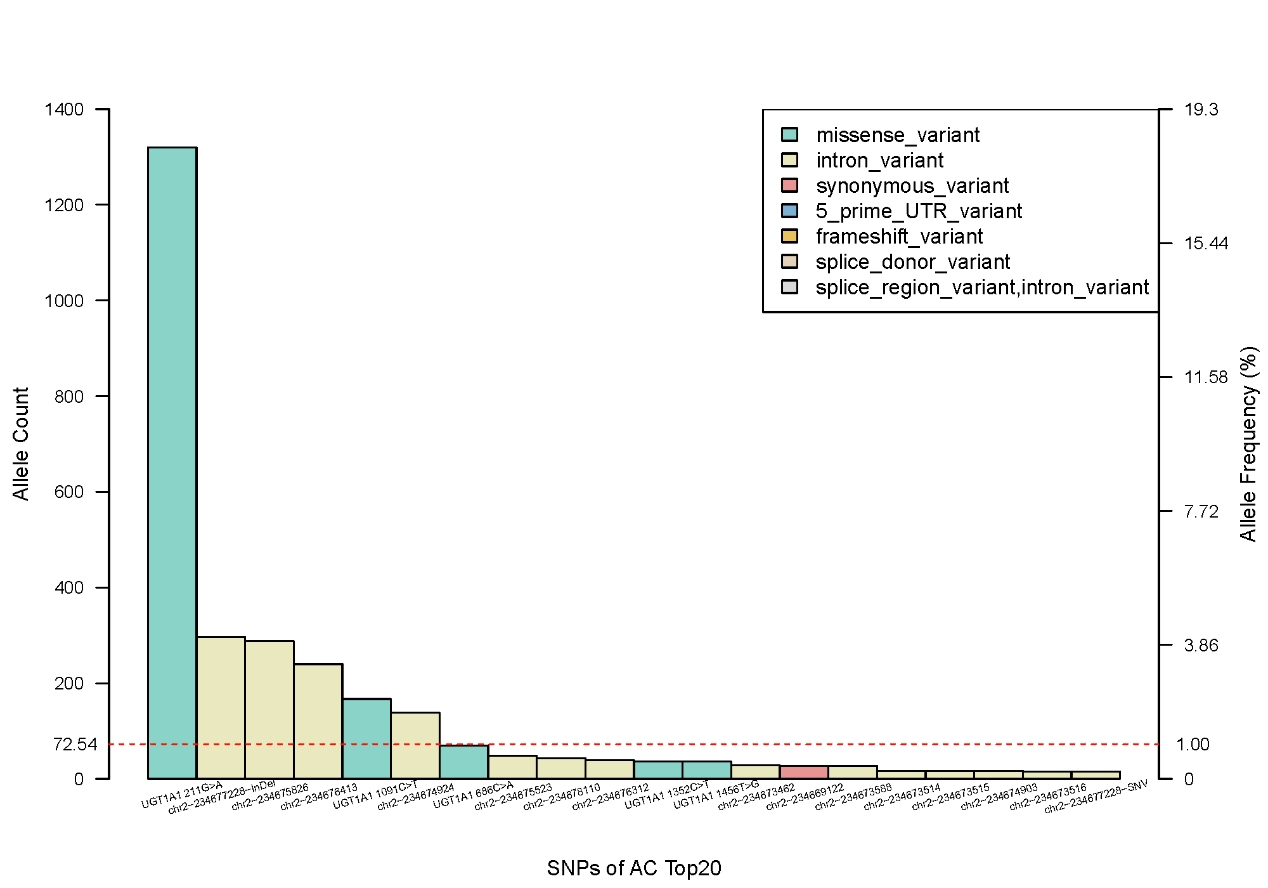


**supFigure1.** Allele counts (the left x-axis) and frequencies (the right x-axis) of top 20 of *UGT1A1* polymorphisms. Different color denotes specific predicted effects of each allele on its corresponding canonical transcript annotated by VEP.


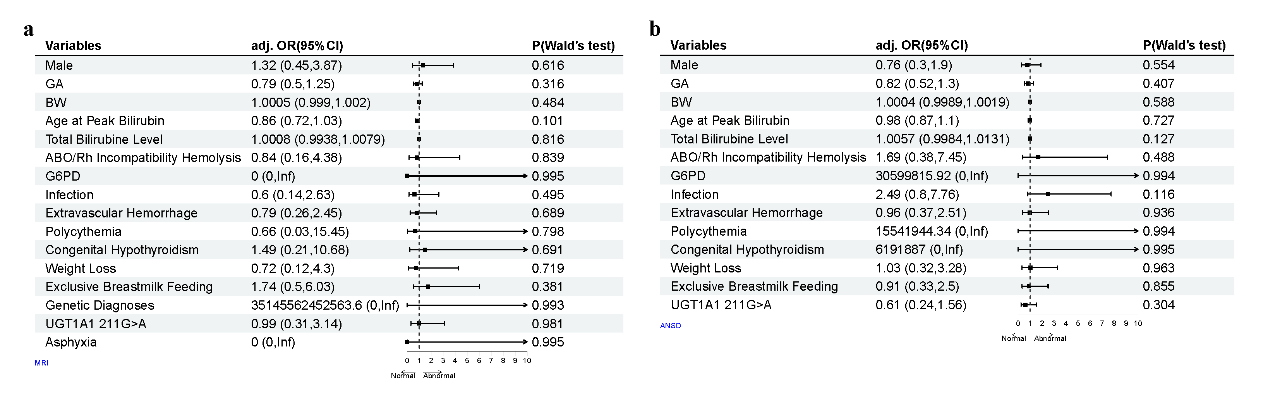


**supFigure2.** Forest plot of the risk factors for severe hyperbilirubinemia related the abnormality of magnetic resonance imaging and auditory neuropathy spectrum disorder. Multivariate logistic regression analyses between the SHB and control groups were conducted using binomial generalized linear models (glm) and described as odds ratio (OR) with 95% confidence intervals (CIs). All statistical tests were two-tailed, with a significance level at 0.05.

**a.** Forest plot of the risk factors for severe hyperbilirubinemia related the abnormality of magnetic resonance imaging.

**b.** Forest plot of the risk factors for severe hyperbilirubinemia related the auditory neuropathy spectrum disorder.
